# Lysophospholipid stereoisomers exert distinct GPR55-mediated functions *via* different Gα subunits

**DOI:** 10.1016/j.jbc.2025.110324

**Published:** 2025-05-30

**Authors:** Adam T. Guy, Yuta Kohro, Koki Kano, Xianyue Huang, Linchi Chen, Noriko Ooashi, Mariko Inoue, Nozomi Ishii, Tomohiro Yamashita, Yoshio Hirabayashi, Itaru Imayoshi, Ichiro Matsuo, Peter Greimel, Makoto Tsuda, Hiroyuki Kamiguchi

**Affiliations:** 1 Laboratory for Neural Cell Dynamics, RIKEN Center for Brain Science, Wakoshi, Saitama, Japan; 2 Graduate School of Biostudies, Kyoto University, Sakyo-ku, Kyoto, Japan; 3 Department of Molecular and System Pharmacology, Graduate School of Pharmaceutical Sciences, Kyushu University, Higashi-ku, Fukuoka, Japan; 4 Graduate School of Science and Technology, Gunma University, Kiryushi, Gunma, Japan; 5 RIKEN Cluster for Pioneering Research, Wakoshi, Saitama, Japan; 6 Institute for Life and Medical Science, Kyoto University, Sakyo-ku, Kyoto, Japan; 7 Center for Living Systems Information Science, Sakyo-ku, Kyoto, Japan

**Keywords:** axon, chemotaxis, G protein–coupled receptor, lysophospholipid, stereoselectivity

## Abstract

Vertebrate glycerophospholipids typically exhibit a glycerol-3-phosphate (G3P)–configured backbone corresponding to the *R*-configured stereoisomer at the *sn*-2 chiral center. We previously found that the lysoglycerophospholipid *lyso*-phosphatidyl-β-d-glucoside (LysoPtdGlc) with G3P configuration, *R*-LysoPtdGlc, is an endogenous ligand of the G protein–coupled receptor GPR55, acting as an axon guidance cue *via* GPR55–Gα_13_ signaling. However, LysoPtdGlc is hydrolytically derived from phosphatidyl-β-d-glucoside (PtdGlc), which exists *in vivo* in a 6:1 mixture of two stereoisomers, G3P-configured *R*-PtdGlc and glycerol-1-phosphate–configured *S*-PtdGlc. To test whether the stereoconfiguration of LysoPtdGlc influences its biological activity, we combined molecular dynamics simulations of GPR55 activation by *R*-LysoPtdGlc and *S*-LysoPtdGlc with *in vitro* and *in vivo* biological assays of GPR55-mediated functions in nervous system. Molecular dynamics simulations predicted that *R*-LysoPtdGlc, but not *S*-LysoPtdGlc, remained in the putative ligand-binding pocket of the GPR55–Gα_13_ complex. Utilizing our previously established synthetic access to *R*-LysoPtdGlc and *S*-LysoPtdGlc, we investigated *in vitro* axonal chemotropic responses to these two stereoisomers. We observed *R*-LysoPtdGlc-mediated chemorepulsion corroborating our previous studies, and unexpectedly, *S*-LysoPtdGlc-induced chemoattraction *via* GPR55–Gα_S_. Since these phenomena were observed in nociceptive neurons, we tested whether intrathecal administration of *R*-LysoPtdGlc or *S*-LysoPtdGlc induced a nociceptive phenotype in adult mice and found that *R*-LysoPtdGlc but not *S*-LysoPtdGlc increased behavioral sensitivity to mechanical stimuli, and that this response was dependent on GPR55. These data indicate that the stereoconfiguration of LysoPtdGlc determines its biological activity and suggest, at least *in vitro*, that LysoPtdGlc stereoisomers exert distinct GPR55-mediated functions *via* different Gα subunits.

Recent advances in pharmacology have updated the traditional two-state model of receptor activation proposed by Black and Leff (1). Today, it is widely recognized that ligands can be selective: they can bind to the same receptor but elicit different cellular responses by activating distinct downstream pathways (2) or by inducing one of several distinct receptor activation states (3). This was initially described in G protein–coupled receptors (GPCRs) as differential activation of β-arrestin signaling over that mediated by Gα proteins (4) or the “preference” of receptors for certain agonists over others, according to the model of agonist trafficking (5). More recent research demonstrated that some ligands can induce separate intracellular downstream pathways by coupling to different Gα protein subunits, a phenomenon observed in many GPCRs (6) including CB1 and CB2 cannabinoid receptors (7), α_2A_-adrenoceptor (8), β_2_-adrenoceptor (9), dopamine D_2_ receptor (10, 11), and neurotensin receptor type 1 (12). In addition to other mechanisms such as receptor heterodimerization, this is a confounding factor in drug development and sometimes can explain why apparently receptor-specific candidate drugs can produce inconsistent or even contradictory effects depending on the assay or model used (13).

GPR55 is a class A (rhodopsin-like) GPCR expressed in nervous system (14) and other organs (15). Despite GPR55 being implicated in multiple disease states (16), no therapeutic drug specifically targeting GPR55 has been developed yet. GPR55 was originally described as a cannabinoid receptor (17), but subsequently, it was reported that GPR55 is activated by the lysoglycerophospholipids, lysophosphatidylinositol (18) and *lyso*-phosphatidyl-β-d-glucoside (LysoPtdGlc) (19). Of the natural ligands of GPR55 published to date, we reported LysoPtdGlc to have the greatest affinity *in vitro* and identified a LysoPtdGlc–GPR55 signaling mechanism *in vivo* (19). We found that LysoPtdGlc is a glial-derived axon guidance cue in spinal cord sensory circuit development, which induces chemorepulsion in nociceptive afferents *via* LysoPtdGlc activation of GPR55–Gα_13_–Rho–ROCK pathway (19).

Isomerism delineates compounds with identical chemical formulae but distinct chemical and physical properties. Stereoisomers, one subgroup of isomers, are defined as compounds with identical sequence of bonded atoms but possessing different three-dimensional structures. Differences in three-dimensional structures are caused by the presence of asymmetric atoms referred to as chiral centers, which are atoms such as carbon or phosphorus that are bound to four different functional groups. Stereoisomers are further divided into two main subsets: enantiomers, isomers that are mirror images of each other, and diastereomers, non–mirror image isomers. The major classes of biological macromolecules and their building blocks, such as DNA, RNA, proteins, nucleic acids, carbohydrates, and lipids, all feature chiral centers. Enantioselective recognition represents a fundamental principle governing the differential interaction between chiral molecules, including protein–ligand interaction. Glycerophospholipids are a class of lipids whose structure is based on a glycerol backbone bound to two fatty acid chains and a phosphodiester-linked polar headgroup. Eukaryotic and bacterial glycerophospholipids feature an *sn*-glycerol-3-phosphate (G3P) backbone linking the polar headgroup to their nonpolar hydrocarbon tails, whereas in contrast, archaeal glycerophospholipids utilize an enantiomeric *sn*-glycerol-1-phosphate (G1P) backbone (20). However, in our previous work, we made the unusual discovery that the biological precursor of the lysoglycerophospholipid LysoPtdGlc, phosphatidyl-β-D-glucoside (PtdGlc), is present in fetal rat brain as a 6:1 mixture of G3P:G1P (21, 22). Of the two LysoPtdGlc diastereomers (Fig. 1*A*) derived from G3P-based PtdGlc (*R*-PtdGlc) and G1P-based PtdGlc (*S*-PtdGlc), it was *R*-LysoPtdGlc from the dominant G3P-based PtdGlc that acts as a chemorepulsive cue *via* GPR55–Gα_13_ signaling (19).

**Figure 1:**
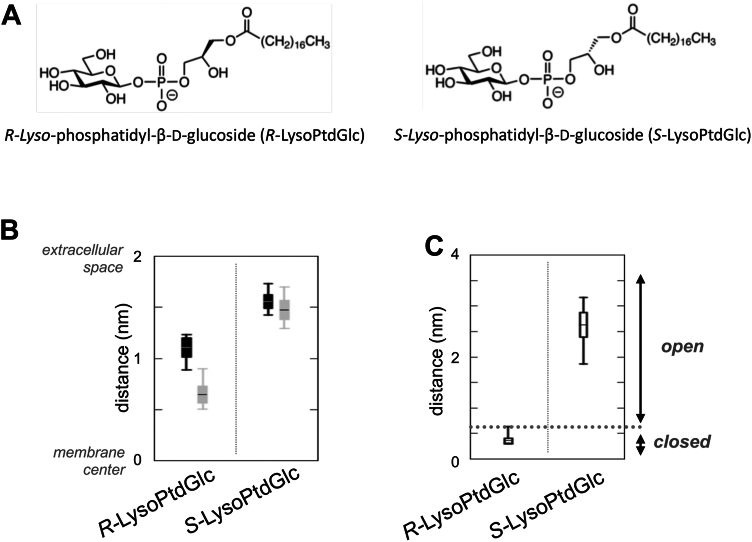
***R*-LysoPtdGlc and *S*-LysoPtdGlc are stereoisomers with differential GPR55-interacting properties.***A*, chemical structures of diastereomers *R*- and *S*-LysoPtdGlc. *B* and *C*, molecular dynamics simulations of GPR55 embedded in a pSM/Chol = 1:1 membrane with *R*-LysoPtdGlc and *S*-LysoPtdGlc ligand. *B*, average ligand position during production run relative to membrane center. Increased distance from the membrane center symbolizes a low binding affinity as the ligand moved out of the putative binding pocket toward the extracellular space; *black*, head group position; *gray*, terminal methyl group of fatty acid residue. *C*, average distance between Asp13 (N terminus) and Lys165 (extracellular loop 2) of the putative ligand entry port. A distance smaller than 0.6 nm (*gray dotted line*) suggests the presence of a salt bridge, indicative of a closed ligand entry port, whereas an increased distance constitutes an open state of the ligand entry port. LysoPtdGlc, *lyso*-phosphatidyl-β-d-glucoside.

In the present study, we examined biological activities of G1P-based LysoPtdGlc (*S*-LysoPtdGlc) in comparison with *R*-LysoPtdGlc and showed that the stereoconfiguration of LysoPtdGlc affects its biological signaling *via* GPR55.

## Results

As a first step to assess whether the G1P-based *S*-LysoPtdGlc and G3P-based *R*-LysoPtdGlc potentially exhibit similar interaction with GPR55, we performed molecular dynamics (MD) simulations. Since structural information on GPR55 remains limited, the previously established GPR55 homology model featuring an active R∗ conformation of the intracellular loop region interacting with helix 5 of Gα_13_ was used (23). While eukaryote-type *R*-LysoPtdGlc remained well inside the putative binding pocket of GPR55 over the course of the unrestrained production run, *S*-LysoPtdGlc stereoisomer exhibited a vertical movement away from the membrane center toward the extracellular space (Fig. 1*B*). This was accompanied with the opening of the putative ligand entry port indicated by the increased distance between D13 and K165 located at the N-terminal region and extracellular loop 2, respectively (Fig. 1*C*). Taken together, these MD results suggest that *S*-LysoPtdGlc exhibits a lower affinity toward GPR55 coupled with Gα_13_, compared with *R*-LysoPtdGlc.

The high similarity of the chemical and physical properties of *R*-LysoPtdGlc and *S*-LysoPtdGlc obstructs their successful separation in extracts of biological tissues, precluding their individual detection and quantification *in vivo*. In order to test the biological activities of the two diastereomers individually *in vitro* and *in vivo*, we therefore opted to use synthetic *R*-LysoPtdGlc and *S*-LysoPtdGlc. The syntheses of *R*-LysoPtdGlc and *S*-LysoPtdGlc were accomplished following our previous reported route (24) by condensation of the benzyl-protected H-phosphonate glucose headgroup with the respective enantiomerically pure *R*-glycerol or *S*-glycerol backbone building block (Fig. S1). Selective deprotection of the naphthyl ether was followed by introduction of stearic acid to the remaining primary hydroxyl function of the glycerol moiety. Final deprotection under reducing conditions gave enantiopure *R*-LysoPtdGlc and *S*-LysoPtdGlc, respectively. The NMR spectra of the obtained *R*-LysoPtdGlc and *S*-LysoPtdGlc were consistent with previous reports (23, 24).

**Figure 2:**
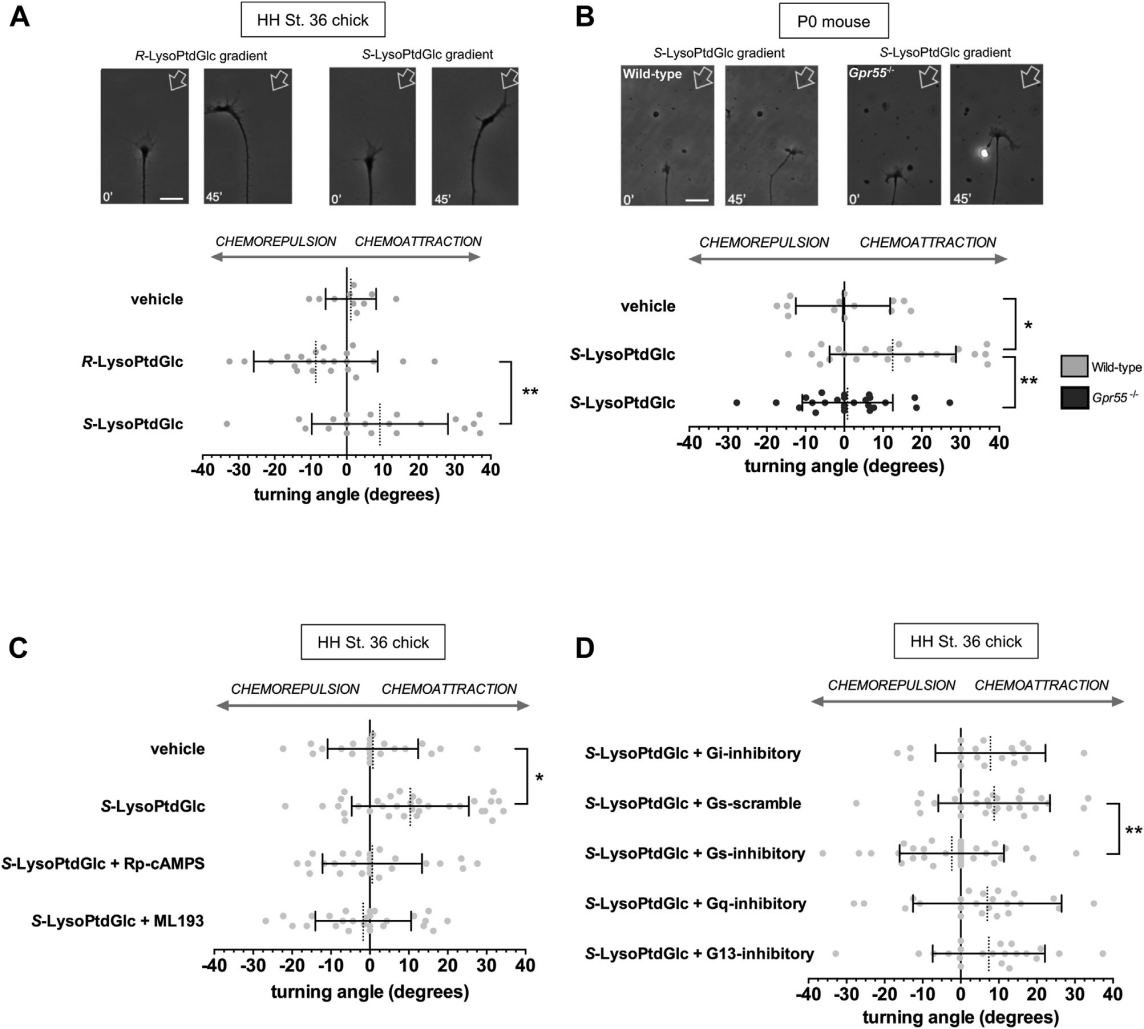
***S*-LysoPtdGlc-induced chemoattraction is dependent on GPR55, adenylyl cyclase/cAMP pathway, and Gα_S_.***A*, representative images of chemorepulsion induced by concentration gradients of *R*-LysoPtdGlc (*left panels*) and chemoattraction induced by *S*-LysoPtdGlc (*right panels*) in Hamburger and Hamilton stage (HH St.) 36 chick sensory neuron axons. *Numbers* indicate time after induction of concentration gradient, and *arrows* indicate the source of gradient. Scale bar represents 10 μm. *Graph* shows quantification of axon turning assay data. *Bars* and *broken vertical line* represent mean ± SD turning angle. Each *gray circle* represents one individual axon tested. ∗∗*p* < 0.01, one-way ANOVA with Tukey’s multiple comparisons post-test. *B*, representative images of chemotropic responses to a concentration gradient of *S*-LysoPtdGlc (source indicated by *arrow*) of wildtype (*left panels*) and *Gpr55*^−/−^ (*right panels*) mouse sensory neuron axons. Scale bar represents 10 μm. *Graph* shows quantification of axon turning assay using wildtype or *Gpr55*^−/−^ postnatal day 0 (P0) mouse neurons. *Bars* and *broken vertical line* represent mean ± SD turning angle. Each *dark gray circle* represents one individual *Gpr55*^−/−^ neuronal axon tested, and *gray circles* represent wildtype. ∗*p* < 0.05, ∗∗*p* < 0.01, One-way ANOVA with Dunnett’s post-test. *C*, pharmacological inhibition of adenylyl cyclase/cAMP pathway using Rp-cAMPS or inhibition of GPR55 using ML193 abolishes the chemoattractive response to *S*-LysoPtdGlc. *Bars* and *broken vertical line* represent mean ± SD turning angle. Each *gray circle* represents one individual chick sensory neuron axon tested. ∗*p* < 0.05, one-way ANOVA with Dunnett’s post-test. *D*, specific inhibition of Gα_S_, but not Gα_q_, Gα_i_, or Gα_13_, abolishes the chemoattractive response to *S*-LysoPtdGlc. *Bars* and *broken vertical line* represent mean ± SD turning angle. Each *gray circle* represents one individual chick sensory neuron axon tested. ∗∗*p* < 0.01, Kruskal–Wallis test with Dunn’s multiple comparisons post-test. LysoPtdGlc, *lyso*-phosphatidyl-β-d-glucoside.

Since our *in silico* data strongly suggested differential interaction with GPR55, we next compared the chemotropic activities of synthetic *R*-LysoPtdGlc and *S*-LysoPtdGlc in a biological assay using embryonic chick primary sensory neurons that endogenously express GPR55 (19). In contrast to our previous report of growth cone chemorepulsion induced by *R*-LysoPtdGlc–GPR55–Gα_13_ pathway (19), we found that *S*-LysoPtdGlc induced growth cone attraction (Fig. 2*A*). Consistently, in wildtype P0 mouse sensory neurons, *R*-Lyso-PtdGlc elicited growth cone repulsion *via* Gpr55 in our previous study (19), whereas *S*-LysoPtdGlc induced attraction (Fig. 2*B*). We also examined P0 mouse sensory neurons in which Gpr55 was genetically deleted, and these neurons exhibited no positive chemotropic response to a concentration gradient of *S*-LysoPtdGlc, demonstrating that the biological activity of *S*-LysoPtdGlc is specifically mediated by Gpr55 (Fig. 2*B*). Chemotropic responses to *R*-LysoPtdGlc or *S*-LysoPtdGlc did not affect the intrinsic rate of axon growth in chick neurons or mouse neurons (Fig. S2). In embryonic chick neurons, chemoattraction induced by *S*-LysoPtdGlc was attenuated by pretreatment with ML193, a specific inhibitor of GPR55, or Rp-cAMPS, an inhibitor of adenylyl cyclase (Fig. 2*C*). Also in chick neurons, specific inhibition of Gα_S_, but not Gα subunits Gα_13_, Gα_q_, and Gα_i_, abolished the chemoattractive response to *S*-LysoPtdGlc (Fig. 2*D*) but did not alter the rate of axon growth (Fig. S3). We also found that the inhibition of Gα_S_ protein function did not affect chemoattractive or chemorepulsive responses to the GPCR-independent guidance cues nerve growth factor and semaphorin 3A, respectively (Fig. S4), and we have previously established that Gα_S_ does not mediate *R*-LysoPtdGlc activation of GPR55 (19). These results strongly suggest that *S*-LysoPtdGlc induced growth cone attraction by activation of GPR55, the same receptor as *R*-LysoPtdGlc, but signals *via* adenylyl cyclase pathway downstream of Gα_S_ and not Rho–ROCK pathway downstream of Gα_13_. From these data, we conclude that the diastereomers *R*-LysoPtdGlc and *S*-LysoPtdGlc induce polarity-opposite chemotropic responses in axon growth cones by differential pathways downstream of GPR55, suggesting a biased agonism mechanism in our primary cell culture model.

**Figure 3:**
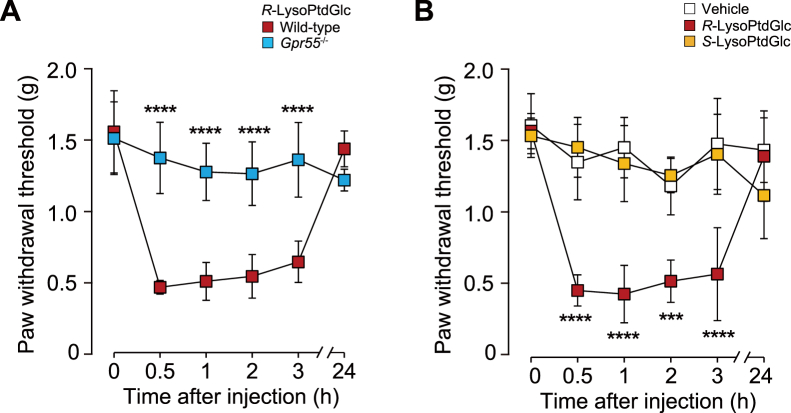
**Intrathecal injection of *R*-LysoPtdGlc but not *S*-LysoPtdGlc induces mechanical hypersensitivity *via* GPR55.***A*, paw withdrawal threshold measured by von Frey test in wildtype or *Gpr55*^−/−^ mice before and after intrathecal injection of *R*-LysoPtdGlc (2.5 nmol) (*n* = 6 mice per group). Data points represent mean ± SD. *B*, paw withdrawal threshold in wildtype mice before and after intrathecal injection of vehicle (5% dimethyl sulfoxide), 2.5 nmol *R*-LysoPtdGlc, or 2.5 nmol *S*-LysoPtdGlc (vehicle and *R*-LysoPtdGlc, *n* = 5 mice; *S*-LysoPtdGlc, *n* = 6 mice). Data points represent mean ± SD. ∗∗∗*p* < 0.001, ∗∗∗∗*p* < 0.0001; repeated-measures two-way ANOVA with Bonferroni’s multiple comparisons test. LysoPtdGlc, *lyso*-phosphatidyl-β-d-glucoside.

To test the hypothesis that *R*-LysoPtdGlc and *S*-LysoPtdGlc possess differential activity at GPR55 *in vivo*, we observed their effect on pain behavior, as previous studies reported that in adult mice, Gpr55 is involved in mechanical pain hypersensitivity (25, 26). We found that intrathecal administration of *R*-LysoPtdGlc significantly decreased the threshold of hindpaw withdrawal in response to light mechanical stimulation in wildtype mice but not in *Gpr55*^−/−^ mice (Fig. 3*A*). However, *S*-LysoPtdGlc administered at the same dose had no effect on the paw withdrawal threshold (Fig. 3*B*). These *in vivo* data are consistent with our *in silico* and *in vitro* findings that the configuration at the *sn*-2 chiral carbon of LysoPtdGlc specifies GPR55-dependent signaling.

## Discussion

In this study, we characterized the biological activities of two structural isomers of the signaling lipid LysoPtdGlc. Based on our previous work (21, 22), we speculated that *R*- and *S*-forms of LysoPtdGlc may possess intrinsically different receptor-activating functions. Beginning with an *in silico* modeling approach, we found that *S*-LysoPtdGlc was predicted to have a very low possibility of activation of GPR55–Gα_13_, in contrast to *R*-LysoPtdGlc. Based on these MD screening results, we next tested the biological activities of *R*- and *S*-LysoPtdGlc using our established axon turning assay, an *in vitro* test of chemotropism using primary cultured nociceptive sensory neurons endogenously expressing GPR55. We found that *S*-LysoPtdGlc induced a positive chemotropic axon growth response, mediated by GPR55 and the adenylyl cyclase pathway downstream of Gα_S_. This unexpected result prompted us to test the effect of *R*- and *S*-LysoPtdGlc *in vivo*. Intrathecal injection of *R*-LysoPtdGlc in adult mice induced a transient hypersensitivity to mechanical stimuli, but injection of *S*-LysoPtdGlc had no such effect. In addition, *R*-LysoPtdGlc-induced mechanical hypersensitivity was not affected by pretreatment with *S*-LysoPtdGlc (Fig. S5).

Functional selectivity of GPR55 has long been speculated to exist both *in vitro* (27) and *in vivo* (28) although there is little direct experimental evidence to date. In an earlier study we identified a role for Gpr55 signaling in modulation of nociceptive somatosensory function in adult mice (26). Here, we found that intrathecal injection of *R*-LysoPtdGlc reduced the paw withdrawal threshold in von Frey test in a Gpr55-dependent mechanism, suggesting that *R*-LysoPtdGlc-mediated activation of Gpr55 may play a role in disorders of nociceptive hypersensitivity. This response was not induced by injection of *S*-LysoPtdGlc, indicating that in this system, the Gpr55-mediated response is stereospecific for G3P-configured *R*-LysoPtdGlc. Considering our *in vitro* turning assay and *in vivo* data together, our results suggest a possible mechanism of functional selectivity of stereoisomers upon activation of GPR55, although the *in vivo* significance of G1P-based *S*-LysoPtdGlc remains to be elucidated.

The phylogenetic tree of life based on small ribosomal subunit gene sequences depicts a closer relationship between the Archaea and Eukarya domains compared with Bacteria. In contrast, eukaryotic and bacterial glycerophospholipids utilize the same G3P-based backbone, compared with Archaea, which instead feature a G1P-configured glycerol backbone (29). The respective biosynthetic enzymes, G3P dehydrogenase and *sn*-glycerol-1-phosphate G1P dehydrogenase, exhibit no sequence similarity strongly suggesting an independent evolutionary origin (30). This difference between the domains remains an unsolved question in evolutionary biology and is referred to as the “lipid divide” (20). Although experiments using transgenic bacteria have demonstrated that a heterochiral mixture of G1P- and G3P-based phospholipids can form stable hybrid membranes (31), they are not found in nature (32). However, the lipid divide is not complete, as *bis*(monoacylglycero)phosphate is a natural G1P-based lipid found in eukaryotic late endosomes and lysosomes (33, 34). Despite recent advances (35), the key step in the *bis*(monoacylglycero)phosphate biosynthetic pathway establishing its exclusive G1P backbone configuration remains to be identified. In contrast, the biosynthesis of PtdGlc, the precursor of LysoPtdGlc, has recently been reported to proceed *via* UDP-glucose:glycoprotein glucosyltransferase–mediated condensation of UDP-glucose with diacylglycerol (DAG) (36). This unusual pathway omits the common glycerophospholipid precursor cytidine diphosphate-DAG with its exclusive G3P configuration in eukaryotes. The reported UDP-glucose acceptor promiscuity of UDP-glucose:glycoprotein glucosyltransferase together with the well-established ester migration in DAG *in vitro* and *in vivo* conceivably rationalizes the reported presence of the 6:1 mixture of G3P:G1P in fetal rat brain tissue (21, 22). New technology may allow us to reliably isolate other G1P-based phospholipids from nervous system tissue and elucidate their functions in development and pathophysiological conditions in the future.

Regarding the synthesis of lysoforms of PtdGlc, the ready cleavage of the *sn*-2 acyl chain located on the *R*-configured central carbon atom of the G3P-based PtdGlc (*R*-PtdGlc) yielding G3P-based LysoPtdGlc (*R*-LysoPtdGlc) by phospholipase A_2_ enzymes has been well documented (37). However, to date, no mammalian phospholipase A_2_ has been reported to be able to cleave the *S*-configured *sn*-2 acyl chain present in G1P-based glycerophospholipids. Nevertheless, no such stereoselectivity is found in members of the phospholipase A_1_ family, which readily cleave the *sn*-1 acyl chain of both G3P- and G1P-based glycerophospholipids. Therefore, we can speculate that conversion of the G1P-based PtdGlc (*S*-PtdGlc) precursor to the G1P-based LysoPtdGlc (*S*-LysoPtdGlc) can be achieved by a phospholipase A_1_ activity releasing the acyl chain at position *sn*-1, followed by nonenzymatic acyl migration (24) from the *sn*-2 to the energetically more favorable primary alcohol at *sn*-1.

In summary, we have identified LysoPtdGlc–Gpr55 signaling as a mechanism of mechanical hypersensitivity in the adult mouse that has high stereoselectivity for the G3P-configured *R*-LysoPtdGlc. However, our *in vitro* data also suggest the possibility of a biological function of *S*-LysoPtdGlc activating GPR55 and adenylyl cyclase pathway downstream of Gα_S_, hinting that specific roles for G1P-based lipids may exist in eukaryotic cells *in vivo*. We speculate that G1P-based lipids in eukaryotic cells may be more common than previously understood, and that these lipids may possess specific biological functions that remain to be elucidated.

## Experimental procedures

### MD simulations

MD simulations were performed under NPT conditions (Langevin piston method, 1.01325 bar, 100 fs oscillating time, and 50 fs damping constant) using NAMD 2.14 (38) and CHARMM36 force field (39). Simulations were performed at 310 K, 2 fs per step, electrostatic interactions were treated using particle-mesh Ewald method, and van der Waals interactions were cutoff at 12 Å. The active-state R∗ homology model of GPR55 featuring a truncated helix 5 of Gα_13_ at the intracellular side and a ligand in its putative binding pocket was embedded in an N-palmitoylsphingomyelin:cholesterol 1:1 membrane patch to mimic raft environment, hydrated with TIP3 water, and neutralized with potassium chloride at 100 mM as reported previously (23). For each probed ligand, the initial position inside the putative binding pocket was kept identical. Each ensemble was equilibrated for 50 ns, and harmonic restraints were reduced stepwise during the initial 2 ns. The equilibration run was followed by a 20 ns production run. The trajectories were analyzed using VMD and proprietary scripts. All graphs were generated with gnuplot 4.

### Animals

All experimental protocols and housing conditions for animals were reviewed and approved by RIKEN’s Wako Animal Experiments Committee, Kyoto University’s Animal Experiment Planning Committee, or Kyushu University’s Institutional Animal Care and Use committee review panel, before the start of the study. All the animals were cared for and treated humanely in accordance with the institutional guidelines for experiments using animals. Fertilized Boris Brown chicken eggs were purchased from a local supplier (Inoue Poultry Farm) and incubated in a humidified rocking egg incubator at 38 °C until the embryos had developed to Hamburger and Hamilton stage 36 (40). Male and female C57BL/6 mice (CLEA Japan) and *Gpr55* knockout mice were used. *Gpr55* knockout mice (B6;129S5-Gpr55^tm1Lex^/Ieg; raised by Lexicon Genetics, Inc) were obtained as heterozygote adults from the European Mouse Mutant Archive and the line maintained in the RIKEN Center for Brain Science animal housing facilities. Offspring mice were genotyped using primers and PCR protocols provided by European Mouse Mutant Archive. Because the *Gpr55* knockout mouse line has a mixed C57BL/6 and 129S5 genetic background, we backcrossed the mice with C57BL/6 mice for 10 generations before use in our experiments. Mouse embryos were staged by the number of days of embryonic development, counting the day of confirmation of vaginal plug as E0.

### Biological assays (*in vitro*)

#### Cell culture

For experiments using chick, nociceptive sensory neurons from dorsal root ganglia (DRG) were extracted from stage 36 chick embryos of either sex and cultured as dissociated cells on laminin-coated dishes as previously described (19). For turning assays using *Gpr55* knockout mice, nociceptive DRG sensory neurons were extracted from postnatal day 0 (P0) pups of either sex and cultured as dissociated cells on laminin-coated dishes as previously described (19).

#### Turning assay

Axon turning assays (41) were performed as we have previously described (19, 23) with some modifications. Before use in the turning assay, 1 mM stock lipid solutions were thawed and diluted to 10 μM in PBS (Life Technologies). Other cues were used at the following in-pipette concentrations: nerve growth factor (Promega) 50 μg/ml and semaphorin 3A (R&D Systems) 10 μg/ml. On an Olympus IX51 inverted microscope (Evident Corporation), a growth cone at the tip of an extending axon was introduced to a microscopic concentration gradient of *R*- or *S*-LysoPtdGlc test compound and imaged for 45 min using a QICAM Fast 1394 (QImaging) CCD digital camera controlled by Metavue software (version 7.8.2.0; Molecular Devices). The microscopic concentration gradient of lipid was produced and maintained by repeated pulsatile ejection of lipid solution through a glass micropipette (borosilicate glass with filament; catalog number: BF100-50-10; Sutter Instrument), the tip of which was placed 100 μm away from the growth cone. Micropipettes were pulled using a P-97 Flaming/Brown micropipette puller (Sutter Instrument), designed to have a steep shoulder and an approximate tip diameter of 1 to 2 μm. Immediately before start of the turning assay, the micropipette was backloaded with 2.5 μl of lipid solution or vehicle using a gel microloader tip. Positive nitrogen gas pressure of 1 to 4 psi was applied to the lipid solution within the micropipette *via* connection to a PV820 Picopump (World Precision Instruments) electrically gated pressure control system. The duration (20 ms) and frequency (2 Hz) of positive pressure was controlled by an electric pulse generator (model AWG-50; ELMOS). The pulsatile ejection of test solution from the pipette induces a microscopic concentration gradient of test compound in the culture medium for the duration of the assay (41, 42). At a distance of 100 μm from the pipette tip, test compound concentration is approximately 1000 times lower than the in-pipette concentration (41, 43). After 45 min, the turning angle (the angle between the growth cone’s start position and its final position, measured relative to its axon) and axon extension, in microns, were calculated using Metavue, as previously described (19). No statistically significant differences were observed in rate of axon extension between isomer test groups (Fig. S2). Growth cones that bifurcated, collapsed, retracted, or failed to extend more than 10 μm were excluded. Experimenter was blind to the diastereomer used in the turning assay experiments in Figure 2, *A* and *B*.

#### Pharmacological agents and peptide inhibitors

Where used, ML-193 (Bio-Techne) or Rp-cAMPS (Merck) were added to the DRG neuron cultures at least 40 min before the start of the turning assay, at a final bath concentration of 10 μM and 20 μM, respectively. Specific inhibitory carboxyl-terminal peptides were used as previously described (19, 44, 45, 46, 47). In brief, inhibitory peptides were designed with the following amino acid sequences: Gα_S_, QRMHLRQYELL (45); Gα_i_, IKNNLKDCGLF (46); Gα_q_, LQLNLKEYNAV (46); Gα_13_, LHDNLKQLMLQ (19, 47); and also a control peptide of scrambled sequence: Gα_S_ (scramble) QLRRYHQELML. Dissociated stage 36 chick DRG neurons were loaded with inhibitory or control peptides by repeated manual trituration in 1 mg/ml peptide solution according to the protocol of Takei *et al.* (48) before being subjected to axon turning assay. No statistically significant differences were observed in rate of axon extension between test groups treated with drugs or inhibitory peptides (Fig. S3).

### Biological assays (*in vivo*)

Male C57BL/6 mice (CLEA Japan), male or female *Gpr55*^−/−^ mice, and their wildtype littermates were used. All mice used were 8 to 12 weeks old at the start of each experiment and were housed at 22 ± 1 °C with a 12 h light–dark cycle with food and water *ad libitum*. Mice were placed individually in an opaque plastic cylinder, laid on a wire mesh, and left for 0.5 to 1 h to allow habituation to the new environment. Then, calibrated von Frey filaments (0.02–2.0 g; North Coast Medical) were applied to the plantar surfaces of the hind paws of mice from below the mesh floor, and the 50% paw withdrawal threshold was calculated using the up–down method (49). Von Frey test was carried out before and after intrathecal injection of *R*-LysoPtdGlc or *S*-LysoPtdGlc. *R*-LysoPtdGlc and *S*-LysoPtdGlc were dissolved in dimethyl sulfoxide and diluted by PBS (the final concentration of dimethyl sulfoxide was v/v 2% or 5%) before use. Intrathecal administration of *R*- or *S*-LysoPtdGlc (2.5 nmol in 5 μl) by injection into the L5/L6 vertebral space using a Hamilton microsyringe connected to a 30-gauge needle was performed as previously described (50). Where both isomers were used simultaneously, *S*-LysoPtdGlc (2.5 nmol in 5 μl) was injected first, and after 30 min, *R*-LysoPtdGlc (2.5 nmol in 5 μl) was then administered.

### Statistics

Statistical analyses were performed using GraphPad Prism (version 6.0c; GraphPad Software, Inc). Experimental datasets were tested for normality by Shapiro–Wilk normality test or D’Agostino and Pearson's omnibus test, and for homoskedasticity using F test or Bartlett’s test. Datasets with normal distribution, and equal variances were analyzed by one-way ANOVA with Tukey’s or Dunnett’s multiple comparisons post-test (Figs. 2, *A*–*C*, and S2). Datasets found not to have normal distribution or equal variance were analyzed using Kruskal–Wallis test with Dunn’s multiple comparisons test (Figs. 2*D*, S3 and S4). Data from behavioral analyses were tested by repeated-measures two-way ANOVA with Bonferroni’s multiple comparisons test (Figs. 3, *A* and *B*, S5). Detailed statistical test data can be provided on request.

## Data availability

All data are contained within the article and supporting information.

## Supporting information

This article contains supporting information (Figs. S1–S5).

## Conflict of interest

The authors declare that they have no conflicts of interest with the contents of this article.

## References

[1] Black J.W., Leff P. (1983). Operational models of pharmacological agonism. Proc. R. Soc. Lond. B Biol. Sci..

[2] Kenakin T. (2007). Functional selectivity through protean and biased agonism: who steers the ship?. Mol. Pharmacol..

[3] Wootten D., Christopoulos A., Marti-Solano M., Babu M.M., Sexton P.M. (2018). Mechanisms of signalling and biased agonism in G protein-coupled receptors. Nat. Rev. Mol. Cell Biol..

[4] Kenakin T.P. (2008). Pharmacological onomastics: what's in a name?. Br. J. Pharmacol..

[5] Kenakin T. (1995). Agonist-receptor efficacy. II. Agonist trafficking of receptor signals. Trends Pharmacol. Sci..

[6] Hermans E. (2003). Biochemical and pharmacological control of the multiplicity of coupling at G-protein-coupled receptors. Pharmacol. Ther..

[7] Glass M., Northup J.K. (1999). Agonist selective regulation of G proteins by cannabinoid CB(1) and CB(2) receptors. Mol. Pharmacol..

[8] Brink C.B., Wade S.M., Neubig R.R. (2000). Agonist-directed trafficking of porcine alpha(2A)-adrenergic receptor signaling in Chinese hamster ovary cells: l-isoproterenol selectively activates G(s). J. Pharmacol. Exp. Ther..

[9] Ghanouni P., Gryczynski Z., Steenhuis J.J., Lee T.W., Farrens D.L., Lakowicz J.R. (2001). Functionally different agonists induce distinct conformations in the G protein coupling domain of the beta 2 adrenergic receptor. J. Biol. Chem..

[10] Kilts J.D., Connery H.S., Arrington E.G., Lewis M.M., Lawler C.P., Oxford G.S. (2002). Functional selectivity of dopamine receptor agonists. II. Actions of dihydrexidine in D2L receptor-transfected MN9D cells and pituitary lactotrophs. J. Pharmacol. Exp. Ther..

[11] Mottola D.M., Kilts J.D., Lewis M.M., Connery H.S., Walker Q.D., Jones S.R. (2002). Functional selectivity of dopamine receptor agonists. I. Selective activation of postsynaptic dopamine D2 receptors linked to adenylate cyclase. J. Pharmacol. Exp. Ther..

[12] Skrzydelski D., Lhiaubet A.M., Lebeau A., Forgez P., Yamada M., Hermans E. (2003). Differential involvement of intracellular domains of the rat NTS1 neurotensin receptor in coupling to G proteins: a molecular basis for agonist-directed trafficking of receptor stimulus. Mol. Pharmacol..

[13] Galandrin S., Oligny-Longpre G., Bouvier M. (2007). The evasive nature of drug efficacy: implications for drug discovery. Trends Pharmacol. Sci..

[14] Sawzdargo M., Nguyen T., Lee D.K., Lynch K.R., Cheng R., Heng H.H. (1999). Identification and cloning of three novel human G protein-coupled receptor genes GPR52, PsiGPR53 and GPR55: GPR55 is extensively expressed in human brain. Brain Res. Mol. Brain Res..

[15] Henstridge C.M., Balenga N.A., Kargl J., Andradas C., Brown A.J., Irving A. (2011). Minireview: recent developments in the physiology and pathology of the lysophosphatidylinositol-sensitive receptor GPR55. Mol. Endocrinol..

[16] Piñeiro R., Falasca M. (2012). Lysophosphatidylinositol signalling: new wine from an old bottle. Biochim. Biophys. Acta.

[17] Ryberg E., Larsson N., Sjogren S., Hjorth S., Hermansson N.O., Leonova J. (2007). The orphan receptor GPR55 is a novel cannabinoid receptor. Br. J. Pharmacol..

[18] Oka S., Nakajima K., Yamashita A., Kishimoto S., Sugiura T. (2007). Identification of GPR55 as a lysophosphatidylinositol receptor. Biochem. Biophys. Res. Commun..

[19] Guy A.T., Nagatsuka Y., Ooashi N., Inoue M., Nakata A., Greimel P. (2015). Glycerophospholipid regulation of modality-specific sensory axon guidance in the spinal cord. Science.

[20] Koga Y. (2014). From promiscuity to the lipid divide: on the evolution of distinct membranes in Archaea and Bacteria. J. Mol. Evol..

[21] Nagatsuka Y., Horibata Y., Yamazaki Y., Kinoshita M., Shinoda Y., Hashikawa T. (2006). Phosphatidylglucoside exists as a single molecular species with saturated fatty acyl chains in developing astroglial membranes. Biochemistry.

[22] Greimel P., Ito Y. (2008). First synthesis of natural phosphatidyl-β-D-glucoside. Tetrahedron Lett..

[23] Guy A.T., Kano K., Ohyama J., Kamiguchi H., Hirabayashi Y., Ito Y. (2019). Preference for glucose over inositol headgroup during lysolipid activation of G protein-coupled receptor 55. ACS Chem. Neurosci..

[24] Kano K., Ishii N., Hirayabashi Y., Kamiguchi H., Greimel P., Matsuo I. (2021). Stereocontrolled synthesis of *lyso*-phosphatidyl-β-D-glucoside. ChemistrySelect.

[25] Gangadharan V., Selvaraj D., Kurejova M., Njoo C., Gritsch S., Skoricova D. (2013). A novel biological role for the phospholipid lysophosphatidylinositol in nociceptive sensitization via activation of diverse G-protein signalling pathways in sensory nerves in vivo. Pain.

[26] Ono T., Yamashita T., Kano R., Inoue M., Okada S., Kano K. (2023). GPR55 contributes to neutrophil recruitment and mechanical pain induction after spinal cord compression in mice. Brain Behav. Immun..

[27] Henstridge C.M., Balenga N.A., Schroder R., Kargl J.K., Platzer W., Martini L. (2010). GPR55 ligands promote receptor coupling to multiple signalling pathways. Br. J. Pharmacol..

[28] Laprairie R.B., Kulkarni P.M., Deschamps J.R., Kelly M.E.M., Janero D.R., Cascio M.G. (2017). Enantiospecific allosteric modulation of cannabinoid 1 receptor. ACS Chem. Neurosci..

[29] Lombard J., Lopez-Garcia P., Moreira D. (2012). The early evolution of lipid membranes and the three domains of life. Nat. Rev. Microbiol..

[30] Koga Y., Kyuragi T., Nishihara M., Sone N. (1998). Did archaeal and bacterial cells arise independently from noncellular precursors? A hypothesis stating that the advent of membrane phospholipid with enantiomeric glycerophosphate backbones caused the separation of the two lines of descent. J. Mol. Evol..

[31] Shimada H., Yamagishi A. (2011). Stability of heterochiral hybrid membrane made of bacterial sn-G3P lipids and archaeal sn-G1P lipids. Biochemistry.

[32] Koga Y. (2011). Early evolution of membrane lipids: how did the lipid divide occur?. J. Mol. Evol..

[33] Brotherus J., Renkonen O. (1974). Isolation and characterisation of bis-phosphatidic acid and its partially deacylated derivatives from cultured BHK-cells. Chem. Phys. Lipids.

[34] Tan H.H., Makino A., Sudesh K., Greimel P., Kobayashi T. (2012). Spectroscopic evidence for the unusual stereochemical configuration of an endosome-specific lipid. Angew. Chem. Int. Ed. Engl..

[35] Medoh U.N., Hims A., Chen J.Y., Ghoochani A., Nyame K., Dong W. (2023). The Batten disease gene product CLN5 is the lysosomal bis(monoacylglycero)phosphate synthase. Science.

[36] Hung H.H., Nagatsuka Y., Solda T., Kodali V.K., Iwabuchi K., Kamiguchi H. (2022). Selective involvement of UGGT variant: UGGT2 in protecting mouse embryonic fibroblasts from saturated lipid-induced ER stress. Proc. Natl. Acad. Sci. U. S. A..

[37] Horibata Y., Nagatsuka Y., Greimel P., Ito Y., Hirabayashi Y. (2007). Sensitivity of phosphatidylglucoside against phospholipases. Anal. Biochem..

[38] Phillips J.C., Braun R., Wang W., Gumbart J., Tajkhorshid E., Villa E. (2005). Scalable molecular dynamics with NAMD. J. Comput. Chem..

[39] Klauda J.B., Venable R.M., Freites J.A., O'Connor J.W., Tobias D.J., Mondragon-Ramirez C. (2010). Update of the CHARMM all-atom additive force field for lipids: validation on six lipid types. J. Phys. Chem. B.

[40] Hamburger V., Hamilton H.L. (1951). A series of normal stages in the development of the chick embryo. J. Morphol..

[41] Lohof A.M., Quillan M., Dan Y., Poo M.M. (1992). Asymmetric modulation of cytosolic cAMP activity induces growth cone turning. J. Neurosci..

[42] Zheng J.Q., Felder M., Connor J.A., Poo M.M. (1994). Turning of nerve growth cones induced by neurotransmitters. Nature.

[43] Ming G.L., Song H.J., Berninger B., Holt C.E., Tessier-Lavigne M., Poo M.M. (1997). cAMP-dependent growth cone guidance by netrin-1. Neuron.

[44] Shpakov A.O. (2011). Signal protein-derived peptides as functional probes and regulators of intracellular signaling. J. Amino Acids.

[45] Gilchrist A., Bunemann M., Li A., Hosey M.M., Hamm H.E. (1999). A dominant-negative strategy for studying roles of G proteins in vivo. J. Biol. Chem..

[46] Gilchrist A., Vanhauwe J.F., Li A., Thomas T.O., Voyno-Yasenetskaya T., Hamm H.E. (2001). G alpha minigenes expressing C-terminal peptides serve as specific inhibitors of thrombin-mediated endothelial activation. J. Biol. Chem..

[47] Rasenick M.M., Watanabe M., Lazarevic M.B., Hatta S., Hamm H.E. (1994). Synthetic peptides as probes for G protein function carboxyl-terminal G alpha s peptides mimic Gs and evoke high affinity agonist binding to beta-adrenergic receptors. J. Biol. Chem..

[48] Takei T., Tokushige N., Cai W., Mikoshiba K. (2000). Indirect visualization of protein loading in living DRG neurons by concomitant trituration loading of fluorescence-labeled dextran. Bioimages.

[49] Chaplan S.R., Bach F.W., Pogrel J.W., Chung J.M., Yaksh T.L. (1994). Quantitative assessment of tactile allodynia in the rat paw. J. Neurosci. Methods.

[50] Hylden J.L., Wilcox G.L. (1980). Intrathecal morphine in mice: a new technique. Eur. J. Pharmacol..

