# Supplementary material for: Lysophospholipid stereoisomers exert distinct GPR55-mediated functions via different Gα subunits
Source: J Biol Chem. 2025 May 30;301(7):110324. doi: 10.1016/j.jbc.2025.110324 (PMC12268648; doi:10.1016/j.jbc.2025.110324)
Supplement: Supplementary Figures [file mmc1.docx]

**Synthesis of *R*-LysoPtdGlc**

***R*-LysoPtdGlc**

**Synthesis of *S*-LysoPtdGlc**

***S*-LysoPtdGlc**

**Supplementary Figure 1:** **Synthesis of *R*-LysoPtdGlc and its stereoisomer *S*-LysoPtdGlc: reagents and conditions**

a) 1) PivCl, pyridine, THF, 0 ℃ to 10 ℃, 2) 0.2M I_2_ solution, 10 ℃ to 25 ℃; b) H_2_NNH_2_^.^H_2_O, MeOH, 40 ℃; c) DDQ, CH_2_Cl_2_/H_2_O = 9/1, 25 ℃; d) stearic acid, EDC, DMAP, CH_2_Cl_2_, 25 ℃; e) H_2_, Pd(OH)_2_/C, 1 eq. AcOH, EtOH, 15 ℃.

**
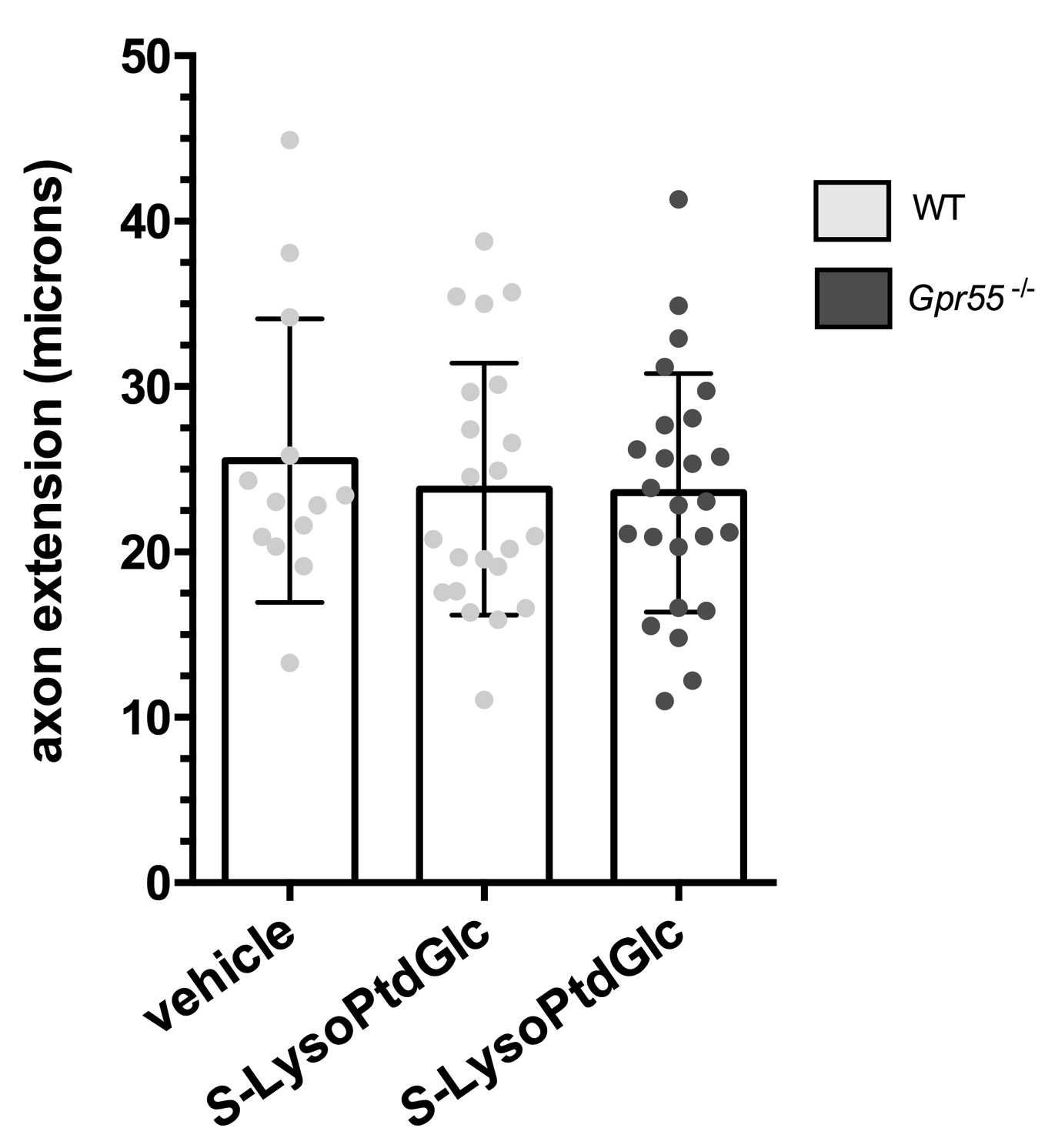

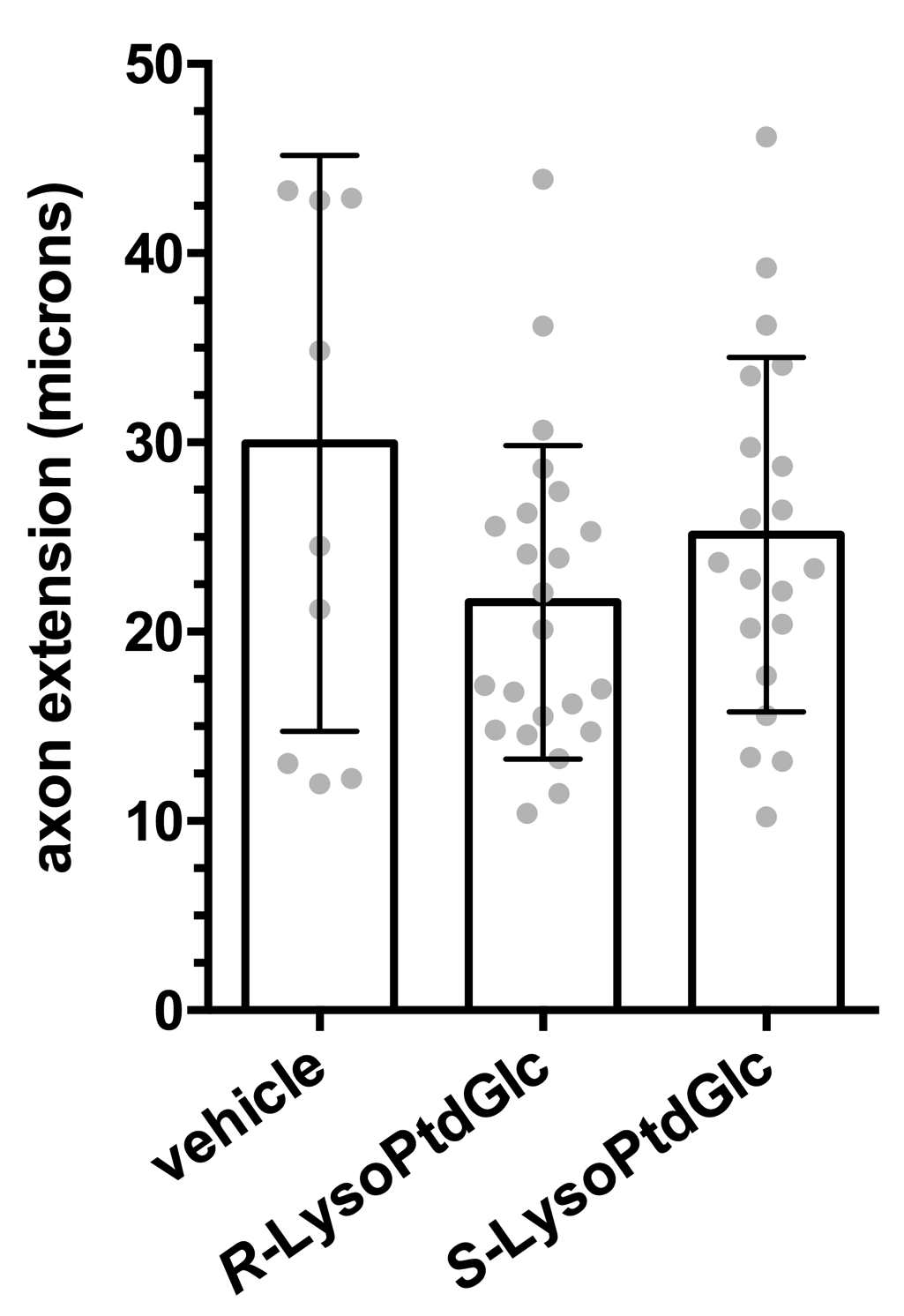
**

P0 mouse

HH St. 36 chick

**Supplementary Figure 2: Rate of axon extension is unchanged during chemotropic responses to *R*-LysoPtdGlc or *S*-LysoPtdGlc in chick or mouse dorsal root ganglion sensory neurons**

No statistically significant difference in mean axon extension was observed in turning assay experiments utilizing either chick (left) or mouse (right) sensory neurons. Bars represent mean ± SD axon extension in microns. Each circle represents one individual axon tested in the axon turning assay. Both datasets were tested by one-way ANOVA with Tukey’s multiple comparisons test.


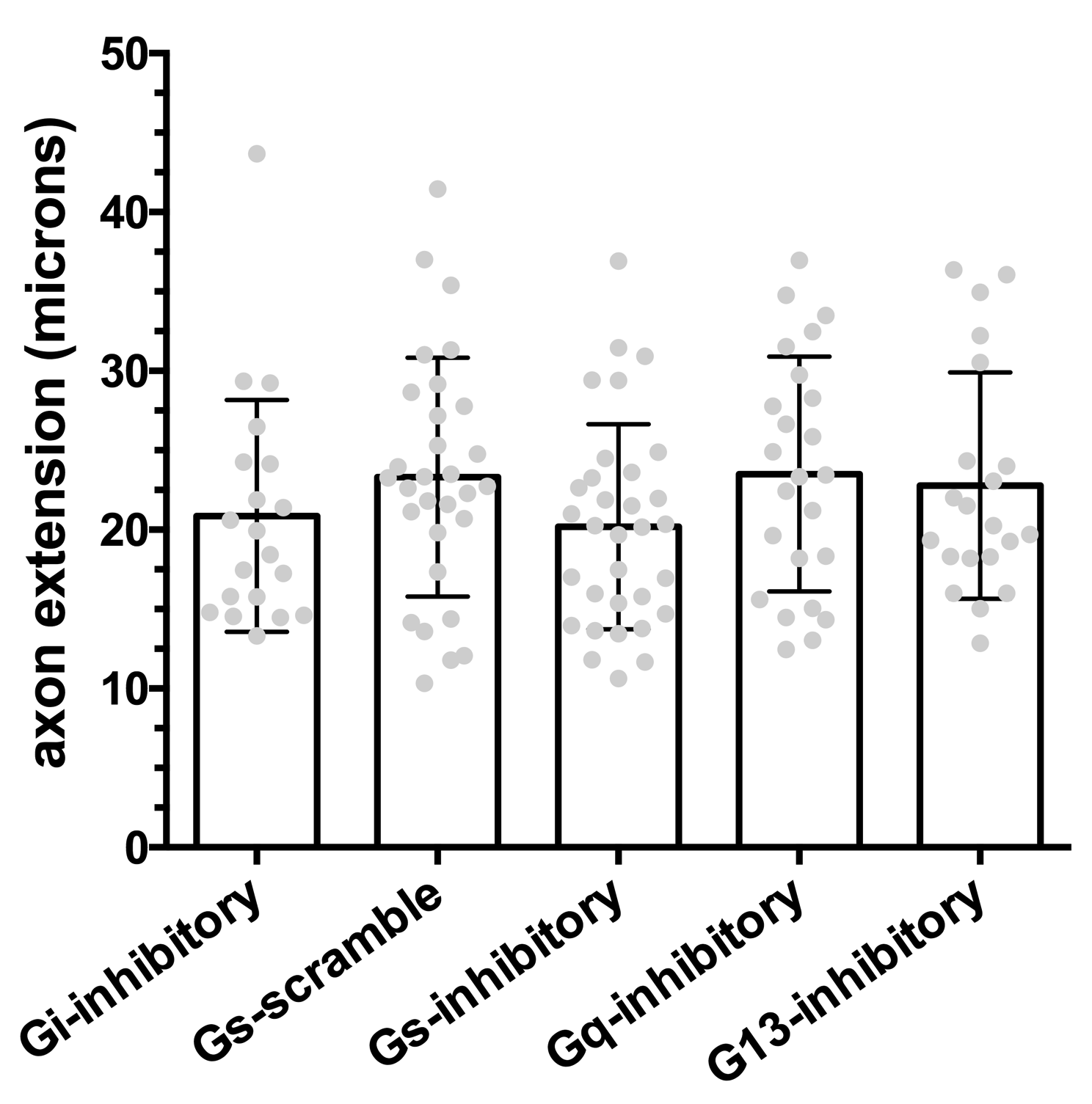
**
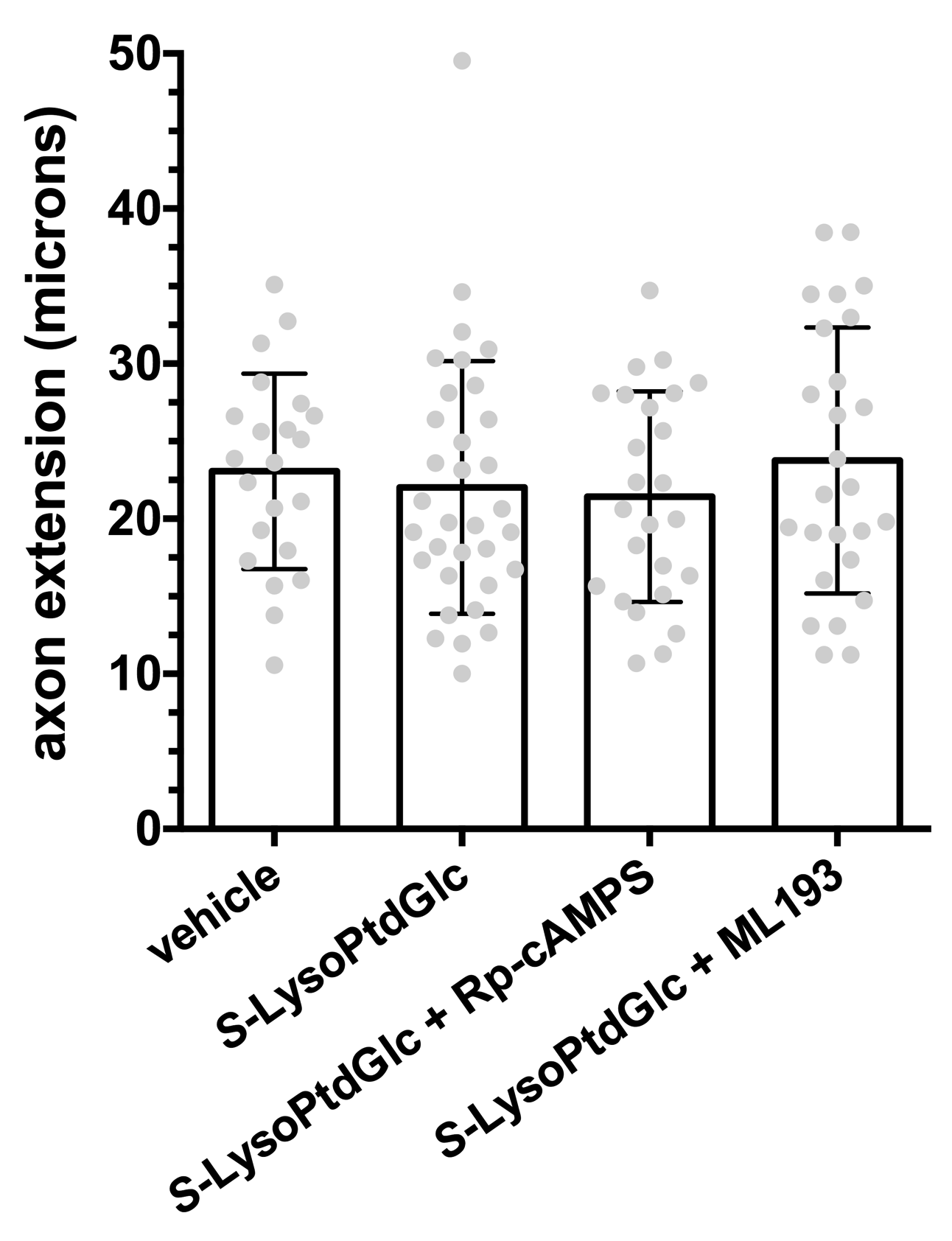
**

HH St. 36 chick

HH St. 36 chick

**Supplementary Figure 3: Axon extension is unaffected by inhibition of adenylyl cyclase pathway with Rp-cAMPs, inhibition of GPR55 by ML193, or by treatment with G protein-inhibitory or scramble peptides**

Treatment with Rp-cAMPS or ML193 (left) or with Gα inhibitory peptides (right) does not affect the rate of axon extension in embryonic chick sensory neurons. Bars represent mean ± SD axon extension in microns. Each gray circle represents one individual axon tested in the axon turning assay. Left: *P* = 0.6844, and right: *P* = 0.2616, Kruskal-Wallis test with Dunn’s multiple comparisons test.

HH St. 36 chick

**
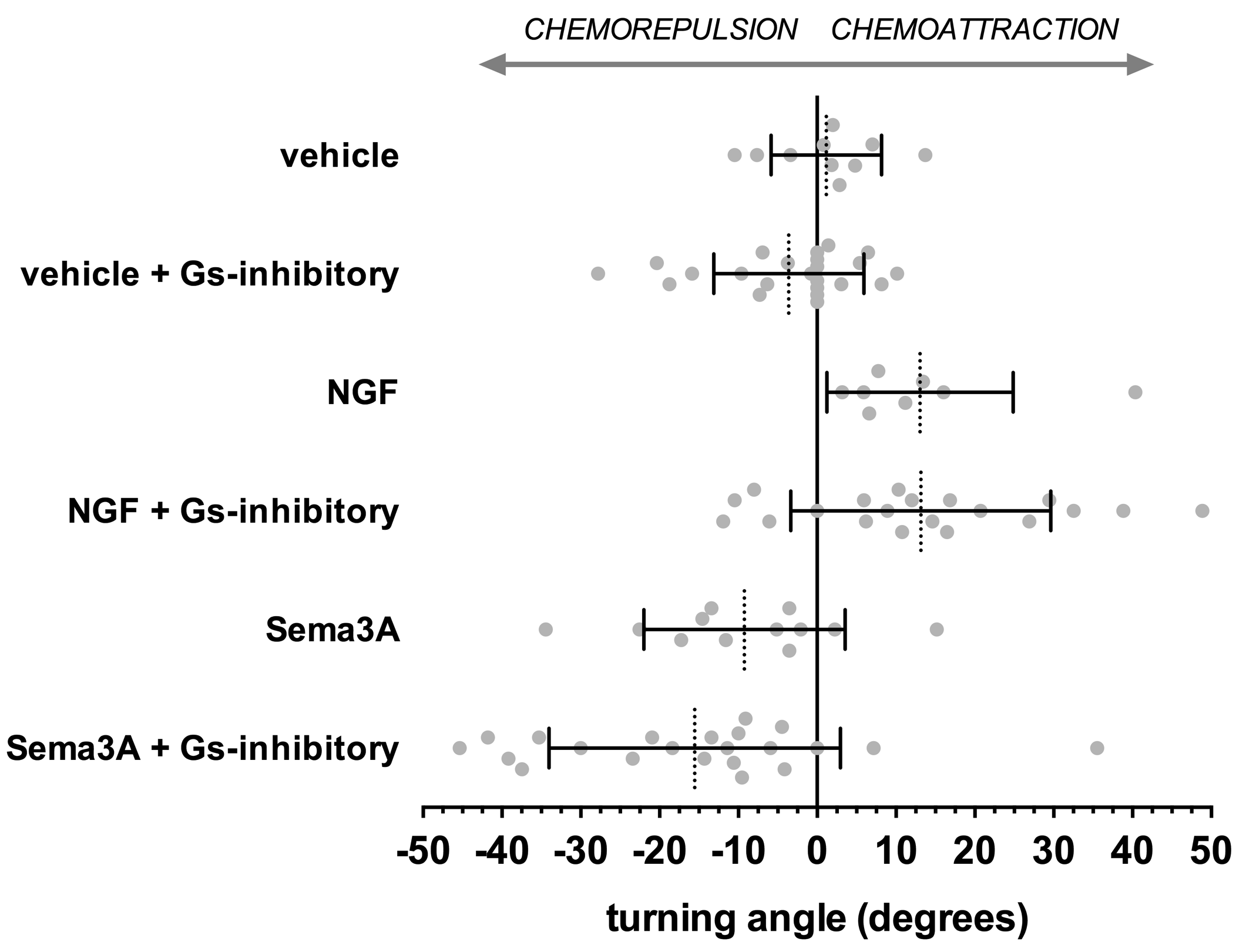
**

**Supplementary Figure 4: Inhibition of Gα_S_ does not affect positive or negative chemotropic axon responses to GPCR-independent guidance cues**

Inhibition of Gα_S_ does not affect embryonic chick sensory neurons’ chemoattractive response to a concentration gradient of nerve growth factor (NGF), or their chemorepulsive response to a gradient of semaphorin A (Sema3A). Bars and broken vertical line represent mean ± SD turning angle. Each gray circle represents one individual axon tested. No statistically significant difference was found between Gα_S_ inhibitory peptide-treated and untreated test groups (Kruskal-Wallis test with Dunn’s post-test). “Vehicle” test group uses the same experimental dataset as shown in Figure 2A.

**
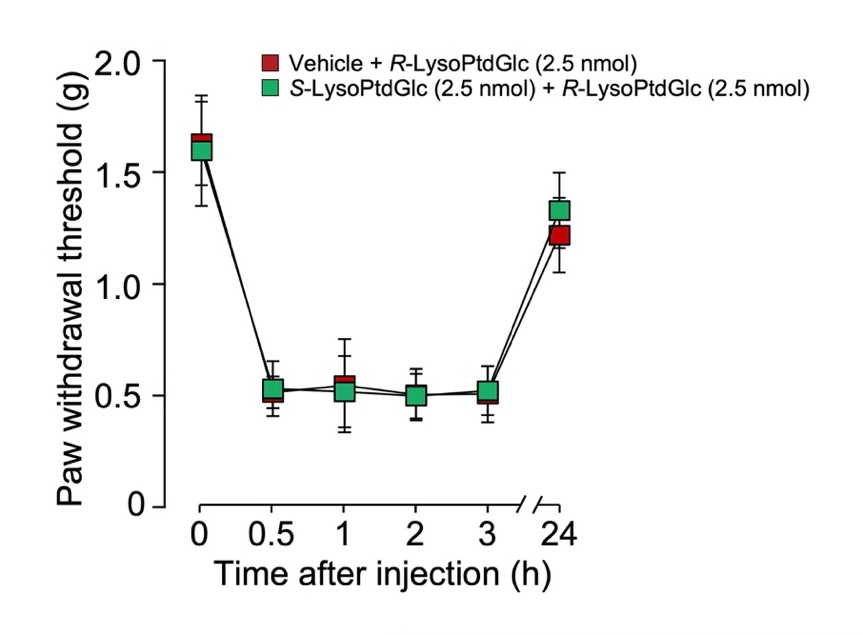
**

**Supplementary Figure 5: Pre-treatment with *S*-LysoPtdGlc does not affect the mechanical hypersensitivity induced by *R*-LysoPtdGlc**

Adult wild-type mice were pre-treated with an intrathecal injection of 2.5 nmol *S*-LysoPtdGlc (green boxes) or vehicle (5% v/v DMSO in PBS; magenta boxes), and after 30 minutes 2.5 nmol *R*-LysoPtdGlc was administered by the same route and subsequently paw withdrawal threshold was measured by von Frey test. Data points represent mean ± SD. No statistically significant difference in withdrawal threshold was observed between vehicle pre-treated (*n* = 5 mice) and *S*-LysoPtdGlc pre-treated animals (*n* = 6 mice).
